# Research Progress on the Prevention and Treatment of Exercise-Induced Fatigue by Acupuncture

**DOI:** 10.3390/healthcare14121734

**Published:** 2026-06-16

**Authors:** Xiaolong Sang, Li Yi, Xu Cai, Yanli You, Xin Wang, Wei Gu

**Affiliations:** 1 Department of Traditional Chinese Medicine, Naval Medical University, Shanghai 200433, China; sxl92445@163.com (X.S.); xiaocaixu@163.com (X.C.); youyanli2005@163.com (Y.Y.); 2 Naval Medical Center, Shanghai 200052, China; yl10120901@163.com

**Keywords:** acupuncture, exercise-induced fatigue, prevention, treatment, mechanism of action

## Abstract

**Highlights:**

**What are the main findings?**

- This review systematically clarifies the multi-target regulatory mechanisms of acupuncture against exercise-induced fatigue, including inhibiting oxidative stress and inflammation, regulating energy metabolism and central nervous system function, and defines the distinct anti-fatigue effects of pre-acupuncture and post-exercise acupuncture interventions.
- Zusanli (ST36) is identified as the core acupoint for acupuncture intervention, and acupoint combinations based on traditional Chinese medicine principles exhibit better synergistic anti-fatigue effects than single acupoint stimulation, with mainstream combination schemes and their functional targets summarized.

**What are the implications of the main findings?**

- The study provides a comprehensive theoretical and research evidence base for the clinical application of acupuncture in preventing and treating exercise-induced fatigue in sports medicine, bridging the research gap between traditional Chinese medicine and modern sports rehabilitation.
- Key limitations of current research are clarified and targeted future research directions are proposed, offering critical guidance for formulating standardized acupuncture intervention protocols and conducting high-quality clinical trials in this field.

**Abstract:**

Exercise-induced fatigue is a common phenomenon after intense exercise or labor, which significantly affects an individual’s exercise performance and physical and mental health. Its timely recovery is crucial for enhancing exercise capacity and preventing injuries. Acupuncture, as a “simple, convenient, inexpensive, and effective” non-pharmaceutical therapy in traditional Chinese medicine, has shown potential unique advantages in the prevention and treatment of exercise-induced fatigue. This narrative review summarizes the recent research progress, with core mechanisms including the inhibition of oxidative stress and inflammation, regulation of energy metabolism, and improvement of central nervous system function, which are mainly verified by preclinical animal studies and partially supported by small-sample clinical trials. The application effects are reflected in the fact that pre-acupuncture before fatigue can enhance anti-fatigue reserves, and post-fatigue treatment can alleviate symptoms and promote recovery, with human evidence limited to small-scale clinical observations. In conclusion, acupuncture has potential therapeutic effects on the prevention and treatment of exercise-induced fatigue, providing a theoretical and practical reference for clinical application.

## 1. Introduction

Exercise-induced fatigue refers to a complex multi-dimensional state where the body’s physiological functions temporarily decline and cannot maintain the predetermined exercise intensity due to intense exercise or prolonged labor. Contemporary exercise science divides it into three interrelated types: metabolic fatigue, neuromuscular fatigue, and perceptual fatigue [1,2]. Exercise-induced fatigue not only leads to decreased exercise capacity, muscle soreness, and persistent fatigue, but is also an important cause of sports injuries. Therefore, the effective prevention and timely recovery of exercise-induced fatigue are of great significance for maintaining athletic performance, preventing injuries, and improving the overall public health. Thus, how to effectively prevent and treat exercise-induced fatigue has always been an important topic in sports medicine and rehabilitation medicine.

Currently, modern intervention methods for exercise-induced fatigue mainly include physical therapy and nutritional supplementation strategies, such as hydrogen-rich gas inhalation, which exerts anti-fatigue effects mainly by scavenging reactive oxygen species and inhibiting peripheral inflammatory response [3,4], oral resveratrol and other dietary supplements, which alleviate fatigue via replenishing energy substrates and reducing skeletal muscle oxidative damage [5,6,7,8]. While these interventions mostly target single peripheral fatigue pathways, acupuncture, as a representative non-pharmaceutical therapy of traditional Chinese medicine, can simultaneously modulate both peripheral and central fatigue pathways with holistic multi-target regulation, showing unique complementary advantages for clinical translation. In recent years, the prevention and treatment of exercise-induced fatigue by traditional Chinese medicine has gradually expanded from single herbal intervention [9,10], to a collaborative intervention model including acupuncture, moxibustion [11], and massage [12], and research hotspots have tended towards the optimization of diversified intervention methods, the selection of specific acupoints, and the explanation of deep-seated mechanisms [13]. Acupuncture, as an important representative of non-pharmaceutical therapies in traditional Chinese medicine, exerts its effects by stimulating specific acupoints on the body surface, achieving the functions of unblocking meridians, regulating qi and blood, and balancing yin and yang. Due to its simplicity of operation, strong adaptability to different environments, and rapid onset, acupuncture has increasingly received attention and application in the prevention and treatment of exercise-induced fatigue. This article will systematically review the research progress of acupuncture in this field, with the aim of providing references for subsequent theoretical exploration and clinical practice.

## 2. Literature Search and Screening Methods

We conducted a standardised literature search and screening for this narrative review in accordance with the PRISMA 2020 guidelines to ensure the transparency and reproducibility of the study selection process. Relevant studies on acupuncture and acupuncture-related therapies for exercise-induced fatigue (EIF) were searched in PubMed, Web of Science, CNKI, Wanfang Database and CBM, with the retrieval time range from January 2016 to January 2026. English search terms included “Acupuncture”, “Electroacupuncture”, “TEAS”, “Transcutaneous electrical acupoint stimulation”, “Acupoint stimulation”, “Pre-acupuncture”, “Exercise-induced fatigue”, “EIF”, “Muscle fatigue”, “Physical fatigue”, “Exercise recovery”, “Anti-fatigue”, “Mechanism of action”, “Molecular mechanism” and “Signaling pathway”; Chinese search terms were 针刺 (Acupuncture), 电针 (Electroacupuncture), 经皮穴位电刺激 (Transcutaneous electrical acupoint stimulation), 穴位刺激 (Acupoint stimulation), 针刺预处理 (Pre-acupuncture), 运动性疲劳 (Exercise-induced fatigue), 肌肉疲劳 (Muscle fatigue), 体力疲劳 (Physical fatigue), 运动恢复 (Exercise recovery), 抗疲劳 (Anti-fatigue), 作用机制 (Mechanism of action), 分子机制 (Molecular mechanism) and 信号通路 (Signaling pathway), using a combination of subject terms and free words for comprehensive retrieval.

**Figure 1:**
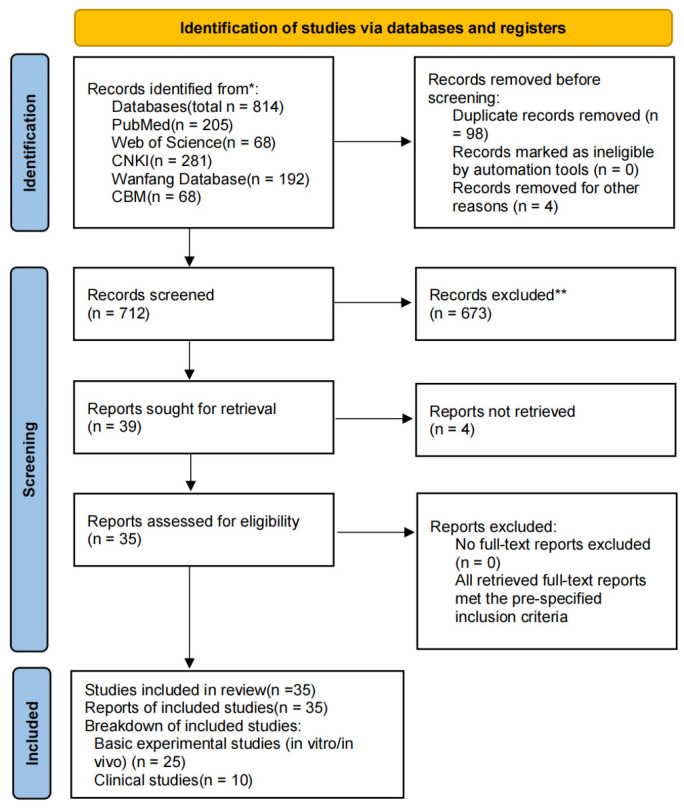
**PRISMA 2020 flow diagram of study selection.** * Databases include PubMed, Web of Science, CNKI, Wanfang Database, and CBM. The retrieval time frame is from January 2016 to January 2026, and a combination of subject terms and free words was used for comprehensive retrieval; ** Reason for exclusion: Irrelevant studies inconsistent with the research topic. Title and abstract screening were completed by independent manual review, no automation tools were used.

Inclusion criteria: original basic experimental studies (in vitro/in vivo), clinical studies, systematic reviews/meta-analyses with complete data, and Chinese/English studies with extractable key data on acupuncture interventions for EIF. Exclusion criteria: duplicate publications, conference abstracts, dissertations, irrelevant studies (e.g., non-acupuncture interventions, non-EIF research), and those with flawed research design. A total of 814 studies were initially retrieved, with 98 duplicates removed and 673 irrelevant ones excluded by title/abstract screening. After retrieving and assessing full-text reports (4 reports not retrieved due to inaccessibility), all retrieved eligible full-text reports met the pre-specified inclusion criteria. Finally, 35 eligible studies were included (25 basic experimental studies and 10 clinical studies, and their detailed characteristics are summarized in Table 1 and Table 2; the literature screening process is presented in Figure 1), covering the anti-EIF mechanisms of acupuncture-related therapies (e.g., inhibiting oxidative stress, regulating energy metabolism, modulating central nervous system function) and the application effects of pre-acupuncture and post-exercise acupuncture interventions. 

## 3. Traditional Chinese Medicine’s Understanding of Exercise-Induced Fatigue

Although exercise-induced fatigue is not directly defined in ancient Chinese medical texts, related discussions have a long history. Its symptoms and pathogenesis can be classified under the categories of “labor fatigue” and “debility”. The statement in “The Yellow Emperor’s Inner Classic” [49] that “excessive labor consumes qi” reveals the core pathogenesis of excessive physical exertion leading to the depletion of essence and qi. “The Golden Cabinet [50]: Blood Stasis and Debility” first explicitly mentioned the term “fatigue”, and Zhang Zhongjing summarized the causes of debility into three types: physical labor, sexual labor, and mental labor. Modern exercise-induced fatigue is mainly caused by excessive physical labor; thus, it can be classified under the category of “physical labor” [51]. From the perspective of organ function theory in Traditional Chinese Medicine (TCM), exercise-induced fatigue is most closely related to the spleen, liver, and kidney. In TCM theory, the spleen is the source of qi and blood production. Excessive physical exertion easily impairs the spleen and stomach, leading to insufficient qi and blood generation and malnutrition of muscles. This then presents with symptoms such as limb weakness and muscle soreness. The liver stores blood and governs the tendons. Excessive exercise depletes liver blood, leaving the tendons and muscles without adequate nourishment. This pathological change can trigger stiffness or injury of the tendons and muscles. The kidneys store essence and govern the bones. Long-term intense exercise depletes the essence and qi of the kidneys, which commonly manifests as symptoms including soreness and weakness of the waist and knees, as well as listlessness. In addition, TCM constitution theory is closely associated with the occurrence of fatigue. Individuals with different TCM constitutions have varying susceptibility to fatigue [52]. Research shows that people with imbalanced constitutions such as qi stagnation, phlegm-dampness, atopic, yin deficiency, and qi deficiency experience more severe fatigue than those with balanced constitutions under the same exercise intensity [10], which reflects the theory of “treating according to individual conditions” in traditional Chinese medicine.

## 4. Analysis of the Mechanism of Acupuncture Therapy in Preventing and Treating Exercise-Induced Fatigue

**Figure 2:**
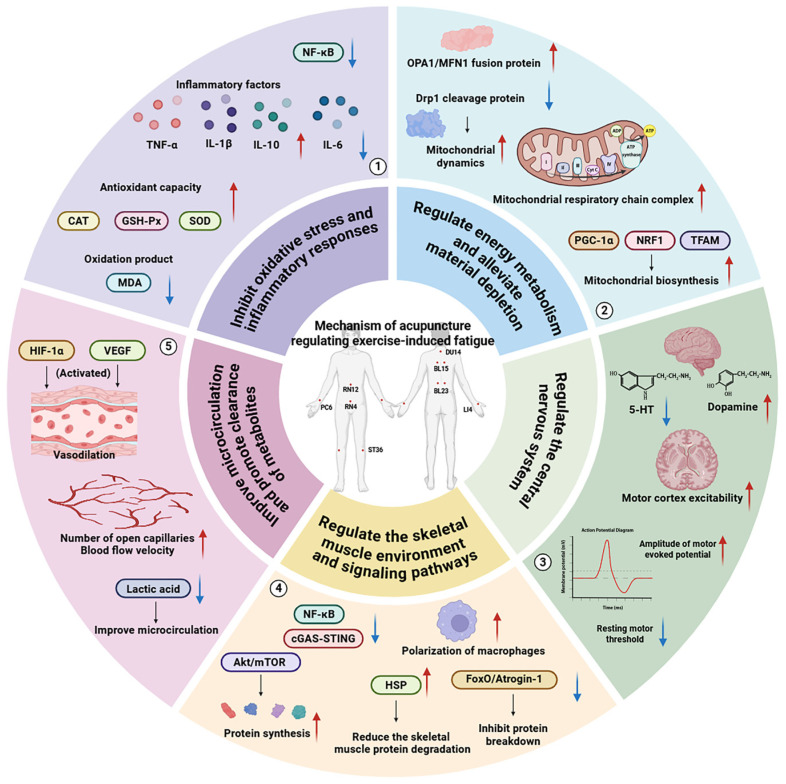
**Mechanisms of Acupuncture in Preventing and Treating Exercise-Induced Fatigue.** This figure illustrates the multi-target and multi-dimensional anti-fatigue effects of acupuncture via stimulation of specific acupoints (including ST36, PC6, RN12, RN4, DU14, BL15, BL23, LI4), where ↑ indicates upregulation, activation or increase, and ↓ indicates downregulation, inhibition or decrease; each numbered section represents an independent regulatory mechanism verified by published studies, with no implication of a unified mechanistic cascade, including ① inhibition of oxidative stress and inflammatory responses via regulating the NF-κB pathway to modulate inflammatory factors (downregulating TNF-α, IL-1β, IL-6 and upregulating anti-inflammatory IL-10), enhance the activity of antioxidant enzymes (SOD, CAT, GSH-Px) and reduce the level of oxidative product MDA, ② regulation of energy metabolism via the PGC-1α/NRF1/TFAM pathway to promote mitochondrial biosynthesis, maintain mitochondrial dynamics (upregulating fusion proteins OPA1/MFN1 and downregulating fission protein Drp1), and increase the expression of mitochondrial respiratory chain complexes to boost ATP production, ③ regulation of the central nervous system via modulating central neurotransmitters (downregulating 5-hydroxytryptamine, 5-HT and upregulating dopamine) and enhancing motor cortex excitability (elevating motor evoked potential amplitude and reducing resting motor threshold) to alleviate exercise-induced central fatigue, ④ regulation of the skeletal muscle microenvironment and signaling pathways via inhibiting NF-κB and cGAS-STING pathways, activating the Akt/mTOR pathway to promote protein synthesis, upregulating heat shock protein (HSP) to reduce skeletal muscle protein degradation, inhibiting the FoxO/Atrogin-1 pathway to suppress protein breakdown, and promoting macrophage polarization to accelerate skeletal muscle repair, and ⑤ improvement of microcirculation and metabolite clearance via activating the HIF-1α/VEGF pathway to induce vasodilation, increase the number of open capillaries and blood flow velocity, and accelerate lactic acid clearance.

Acupuncture, as a classic non-pharmaceutical therapy in traditional Chinese medicine, has demonstrated promising anti-fatigue value in the prevention and treatment of exercise-induced fatigue in recent years. Its effects cover both peripheral aspects, such as the regulation of skeletal muscle energy metabolism, inhibition of oxidative stress, and improvement of microcirculation; and central regulation, including the balance of neurotransmitters, regulation of motor cortex excitability, and maintenance of autonomic nerve function homeostasis. This systematic integrated regulation is achieved via the neuro-endocrine-immune network (Figure 2), with a verified core cascade: peripheral acupoint stimulation is transduced by somatic afferent nerves, which further drives multi-system anti-fatigue regulation through neural, endocrine, and immune pathways [53,54]. Notably, the full continuous causal chain of this network still requires further validation in unified study designs. It achieves systematic integrated regulation through the neuro-endocrine-immune network. The following will elaborate on these five interrelated aspects in detail.

### 4.1. Inhibition of Oxidative Stress and Inflammatory Response

Acupuncture has a significant inhibitory effect on oxidative stress and inflammation. Oxidative stress refers to a state where the body generates a large amount of reactive oxygen species and reactive nitrogen species (RNS) under various stress stimuli, and the generation rate exceeds the body’s antioxidant clearance capacity, leading to an imbalance between oxidation and antioxidation [55]. During high-intensity exercise, the body’s oxygen consumption increases sharply, resulting in the generation of a large number of free radicals. Excessive free radicals can damage the integrity and permeability of biological membranes through lipid peroxidation reactions, causing dysfunction of organelles. The inflammatory response triggered by intense exercise is closely related to oxidative stress, and the levels of pro-inflammatory cytokines (such as TNF-α, IL-1β, IL-6) are consequently elevated [55]. Accumulating evidence has shown that acupuncture can alleviate exercise-induced fatigue-related tissue damage by inhibiting free radical production and regulating related signaling pathways, thereby facilitating fatigue recovery [39]; this effect is closely linked to its core role in suppressing oxidative stress and subsequent inflammatory cascades. Specifically, a study by Ma et al. [14] exploring the effect and mechanism of acupuncture on overloading exercise-induced skeletal muscle injury provided direct experimental evidence for this conclusion. The researchers implemented acupuncture intervention in a rat model of exercise-induced fatigue, and the results showed that the acupuncture group—especially the group receiving the combination of Dazhui (GV14) and Weishu (BL21) points—could significantly reduce malondialdehyde (MDA) content in myocardial and liver tissues and effectively increase the activities of superoxide dismutase (SOD) and glutathione peroxidase (GSH-Px). These findings confir tmhat acupuncture can inhibit exercise-induced oxidative stress responses in rodents by regulating key antioxidant indicators. We explicitly acknowledge inherent species-specific physiological differences between rodents and humans: such preclinical results from rodent mitochondrial and oxidative stress markers cannot be directly generalized to human exercise performance outcomes, though they offer preliminary mechanistic insights for subsequent human clinical research.

Studies have explored the role of oxidative stress regulation in exercise-induced skeletal muscle injury recovery by exogenous supplementation. Melatonin supplementation alone can significantly reduce malondialdehyde (MDA) levels and elevate the activities of antioxidant enzymes, including superoxide dismutase (SOD), glutathione peroxidase (GSH-Px) and catalase (CAT). This result further verifies that targeting oxidative stress is an effective strategy to alleviate exercise-induced muscle injury and accelerate tissue recovery [15]. Yang et al. [16] observed the therapeutic effect and mechanism of the combined acupuncture at Zusanli (ST 36) and Zhongwan (CV 12) on exercise-stress-induced gastric ulcers in rats. They randomly divided 40 male SD rats into a blank group, a model group, an acupuncture group, and an omeprazole group, and measured the levels of serum oxidative stress factors and inflammatory factors in the rats. The results showed that the levels of antioxidant substances SOD and GSH-Px in the serum of the acupuncture group and the omeprazole group were significantly increased, the level of oxidative product MDA was significantly decreased, and the levels of inflammatory factors TNF-α, IL-1β, and IL-6 were significantly decreased, while the level of IL-10 was significantly increased, indicating that acupuncture can mitigate oxidative stress and attenuate inflammatory responses. In addition, the local anti-inflammatory effect mediated by acupuncture involves the regulation of multiple immune cell populations and functions [53]. Studies have indicated that low-intensity electroacupuncture stimulation at the “Zusanli” (ST36) point in mice can specifically activate a group of sensory neurons expressing Prokr2 protein, thereby driving the vagus nerve-adrenal pathway and promoting the release of catecholamines, thus exerting a systemic anti-inflammatory effect [17,56].

### 4.2. Regulation of Energy Metabolism and Alleviation of Material Depletion

The oxidation metabolism of glucose is the main energy source for the human body, and mitochondria are the core of cellular energy metabolism. Acupuncture can alleviate energy supply insufficiency related to exercise-induced fatigue by regulating mitochondrial function and energy metabolism processes. Bai et al. [18] aimed to investigate the time-dependent mitochondrial damage in skeletal muscle after heavy-load exercise and the protective effect of acupuncture on exercise-induced skeletal muscle injury. They randomly assigned 176 male SD rats into four groups: a quiet control group, a sole acupuncture group, a sole exercise group, and an exercise-acupuncture group. The sole acupuncture, sole exercise and exercise-acupuncture groups were further subdivided into different subgroups according to the sample collection time points post-intervention. The results showed that after heavy-load eccentric exercise, the fragmentation of skeletal muscle mitochondria in the exercise-acupuncture group was significantly alleviated, and the protein indicators reflecting mitochondrial status, such as TOMM20/DRP1 and FUNDC1/DRP1, were significantly better than those in the simple exercise group. This indicates that acupuncture intervention can effectively reduce mitochondrial damage caused by heavy-load exercise and promote its functional recovery. Acupuncture exerts a protective effect on mitochondrial function mainly by regulating mitochondrial dynamics. Specifically, it up-regulates the expression of mitochondrial fusion proteins (e.g., OPA1, MFN1) and down-regulates the expression of fission proteins (e.g., Drp1). Thus, it effectively maintains the structural stability and functional integrity of the mitochondrial network [19]. In terms of enhancing energy production, studies have confirmed that acupuncture can promote mitochondrial biogenesis through the PGC-1α/NRF1/TFAM signaling pathway and increase the activity of mitochondrial respiratory chain complexes (such as I, IV, V), thereby significantly increasing the content of adenosine triphosphate in tissues [20]. Adenosine 5′-monophosphate-activated protein kinase (AMPK) is a serine/threonine kinase that acts as a key energy sensor to perceive changes in cellular energy and regulate abnormal energy states. Acupuncture can exert regulatory effects by activating AMPK. Studies have shown that electroacupuncture can significantly promote the phosphorylation (activation) of AMPKα and improve glucose metabolism in the brain, thereby enhancing energy production [21], as an upstream signal, AMPK can positively regulate peroxisome proliferator-activated receptor γ coactivator 1α (PGC-1α), thereby promoting mitochondrial biogenesis and energy supply [22]. Genetic studies have also confirmed that gain-of-function mutations in AMPK can increase mitochondrial content in skeletal muscle, glycogen storage, and significantly enhance fatigue resistance during exercise [23]. Notably, the relationship between AMPK activation and fatigue tolerance is highly context-dependent [57]: Moderate, exercise-induced AMPK activation contributes to cellular energy homeostasis and skeletal muscle fatigue resistance as demonstrated earlier, whereas excessive or sustained activation may impair skeletal muscle force production and contractile function under certain metabolic conditions [58], reflecting AMPK’s dual, context-sensitive regulatory role in exercise metabolism and fatigue. In summary, acupuncture effectively improves energy homeostasis during exercise by regulating mitochondrial function, biogenesis, and key energy metabolism pathways through multiple targets. Acupuncture’s anti-fatigue effect is not limited to peripheral metabolic regulation; it also exerts a central regulatory role by modulating the function of the central nervous system, thus relieving central fatigue induced by exercise.

### 4.3. Regulation of the Central Nervous System

The central nervous system controls and coordinates most of the body’s functions, including movement, sensation, cognition, and the regulation of vital organs. One of the key mechanisms of acupuncture in preventing and treating exercise-induced fatigue lies in its ability to regulate the function of the central nervous system from multiple dimensions, including neurochemistry, neuroelectrophysiology, and the autonomic nervous system [54].

#### 4.3.1. Neurochemical and Behavioral Levels

Acupuncture can improve fatigue-related behaviors by regulating the levels of monoamine neurotransmitters in key brain regions such as the hippocampus, hypothalamus, and striatum. From the perspective of contemporary psychophysiological fatigue models [59], the changes in these neurotransmitters are the core neurochemical basis for regulating perceived exertion (RPE) and central motor drive, which are the key determinants of exercise termination and fatigue perception [60]. Mechanistically, acupuncture (including traditional manual acupuncture, the core intervention of this study, and electroacupuncture) can regulate the function of the hypothalamus and affect the release of neurotransmitters such as dopamine, thereby playing a core role in energy balance and stress response [61]. Specifically, elevated serotonin (5-HT) amplifies central fatigue signals, increases the rating of perceived exertion (RPE), and inhibits descending central motor drive. In contrast, upregulated dopamine reduces exercise-related perceived effort, enhances central motor output, and boosts exercise motivation [24]. Li et al. [25] confirmed that traditional manual acupuncture at Zusanli (ST36) significantly prolonged exhaustive exercise time and reduced hypothalamic 5-HT levels in rats with exercise-induced fatigue, thus providing direct evidence for the role of traditional acupuncture in regulating central neurochemical balance to resist fatigue. In addition, early studies have shown that electroacupuncture can effectively regulate central neurotransmitters in rats with chronic exercise fatigue, such as reducing the levels of serotonin in the hippocampus and hypothalamus and simultaneously improving their exhaustive exercise performance, which serves as important supplementary evidence for the above anti-fatigue mechanism of acupuncture [26]. For electroacupuncture in particular, further research has focused on the specificity of acupoints for electroacupuncture. Recent evidence indicates that electroacupuncture at acupoints such as “Zusanli” can significantly enhance the spontaneous activity level of model animals, and the mechanism is closely related to the regulation of gene expression of serotonin receptors and related hormones in the hypothalamus [27]. Moreover, recent studies have provided more direct pathological morphological correlations in terms of mechanism. Yang et al. [28] found that electroacupuncture intervention in rats with chronic fatigue syndrome improved the pathological morphology of the hippocampus and hypothalamus, such as the arrangement of nerve cells and capillary edema, and significantly increased the number of horizontal and vertical activities of the animals. This result not only verified the effect of electroacupuncture from a behavioral perspective but also directly linked the potential mechanism of neurochemical regulation to the repair of the brain’s microstructure.

#### 4.3.2. Neuroelectrophysiological Level

Acupuncture can regulate the excitability of the motor cortex in the brain. This effect is not limited to electroacupuncture; traditional manual acupuncture also presents similar neuroelectrophysiological regulatory effects. Li et al. [40] conducted a randomized controlled trial, randomly dividing 72 patients with fatigue into an electroacupuncture group and a sham electroacupuncture group. The electroacupuncture group received electrical stimulation at the back-shu points, while the sham electroacupuncture group served as the control. The results showed that compared with the sham electroacupuncture group, the electroacupuncture group had significantly lower FS-14 scores and RMT values, and significantly higher SF-36 scores and MEP-A values. Electroacupuncture at the back-shu points can reduce the resting motor threshold of patients with chronic fatigue syndrome and increase the amplitude of motor evoked potentials, effectively enhancing the excitability of the motor cortex. In the study by Yang et al. [62], it was also confirmed that electroacupuncture at back-shu points such as Xinshu (BL15) and Shenshu (BL23) can significantly enhance the corticospinal excitability of the motor cortex in the lower limb muscle innervation area. In addition, electroacupuncture at upper limb acupoints (such as Hegu (LI4)) has also been shown to produce a sustained excitatory aftereffect on the primary motor cortex of healthy subjects [63], providing supplementary neurophysiological evidence for the improvement of motor control and the alleviation of central fatigue by acupuncture.

#### 4.3.3. Autonomic Nervous System Regulation Level

Disorders of the autonomic nervous system function are one of the important reasons for the persistence of fatigue. Acupuncture can regulate the tension balance between the sympathetic and parasympathetic nerves, thereby improving fatigue caused by such disorders. Heart rate variability (HRV) is an objective indicator for evaluating autonomic nerve function, where high-frequency power mainly reflects parasympathetic nerve activity, while low-frequency power is regulated by both the sympathetic and parasympathetic nerves [64]. Studies have shown that acupuncture exerts its regulatory effect by influencing HRV parameters [65]. A randomized controlled trial conducted by Qing et al. [41] found that acupuncture at the ST36 and CV4 acupoints could effectively regulate HRV parameters in patients with chronic fatigue syndrome (CFS) and improve their fatigue scores. Moreover, the regulatory effect of acupuncture on the autonomic nervous system is acupoint-specific. For instance, acupuncture at the Neiguan acupoint can balance the autonomic nerve by inhibiting the sympathetic nerve center or activating the vagus nerve, thereby regulating heart rate and blood pressure [29]; studies on healthy subjects also indicate that acupuncture at different acupoints (such as ST36 and CV4) can produce differential immediate and sustained regulatory effects on HRV parameters and stress levels [66]. These results suggest that acupuncture can regulate the autonomic nervous system in a specific manner, which may be one of the key mechanisms by which it alleviates various types of fatigue, including exercise-induced fatigue.

On the basis of central and peripheral metabolic regulation, acupuncture further optimizes the skeletal muscle microenvironment by improving local microcirculation, which accelerates the clearance of metabolic wastes and alleviates peripheral fatigue symptoms.

### 4.4. Improving Microcirculation and Promoting Metabolic Waste Clearance

The occurrence of exercise-induced fatigue is closely related to local microcirculation disorders and the accumulation of metabolic wastes in skeletal muscles. Through its traditional Chinese medicine theory of “activating blood circulation and removing blood stasis”, acupuncture plays a crucial role in improving local blood perfusion and accelerating the clearance of metabolic wastes. Firstly, acupuncture can effectively improve local microcirculation and oxygen supply in skeletal muscles. Studies have shown that acupuncture or electroacupuncture stimulation exerts this effect by inducing local vasodilation, increasing the number of open capillaries and elevating blood flow velocity in skeletal muscle tissues [67]. For example, stimulation of the Zusanli acupoint in healthy subjects can significantly increase local blood flow in the area of this acupoint [68]. In exercise-related pathological models, electroacupuncture intervention has also been proven to improve blood supply in ischemic tissues [30]. The underlying mechanism may involve the regulation of vascular-related signaling pathways, e.g., hypoxia-inducible factor 1α (HIF-1α) and Vascular Endothelial Growth Factor (VEGF) [31]. A large number of clinical observations have also confirmed that acupuncture can effectively improve peripheral microcirculation disorders [69].

In exercise models, acupuncture intervention has been shown to alleviate microvascular morphological abnormalities in skeletal muscles caused by high-load exercise, maintain better tissue oxygenation levels, and create a favorable metabolic environment for post-exercise recovery [32]. Secondly, acupuncture can accelerate the clearance of metabolic wastes after exercise: the accumulation of metabolic products such as lactic acid and hydrogen ions caused by high-intensity exercise is a direct cause of peripheral fatigue, and acupuncture can promote local tissue fluid exchange and lymphatic return through mechanical stimulation and neural regulation, thereby accelerating the removal of wastes. A randomized controlled study on sports students confirmed that post-exercise pressurized acupuncture intervention could significantly increase the clearance rate of blood lactic acid, with blood lactic acid concentration approaching resting levels at 30 min [42], indicating the theoretical effect of acupuncture in promoting local circulation and tissue fluid exchange, which helps create favorable conditions for the clearance of metabolic wastes. Additionally, from the perspective of fascia science, the fascia network in skeletal muscles is an important site for microcirculation and material exchange [43]. Acupuncture relieves fatigue-induced myofascial tension and improves muscle compliance, which may restore normal intramuscular pressure gradients and thereby facilitate microcirculation and the physical diffusion of metabolic wastes.

Of note, while enhanced microcirculation has been suggested as a mechanism that aids in clearing metabolic waste, the available evidence is still indirect in nature [33]. The majority of studies have adopted surrogate markers, including blood flow velocity and lactate clearance rate, yet failed to establish a direct association between acupuncture-mediated perfusion alterations and functional performance outcomes such as time to exhaustion and power output. Moreover, given the overreliance on preclinical animal models and the scarcity of high-quality human data, it remains uncertain whether these hemodynamic changes can confer tangible improvements in exercise performance under real physiological conditions. For this reason, the functional relevance of this mechanism deserves a more rigorous critical assessment. In particular, the relative role of peripheral microcirculatory effects in acupuncture’s overall anti-fatigue efficacy—when compared with central regulatory pathways—has not been systematically explored to date [70,71].

### 4.5. Regulating the Intracellular Environment and Signal Pathways in Skeletal Muscles

In addition to regulating systemic energy metabolism and inflammation, acupuncture can directly act on skeletal muscle cells, modulating their internal microenvironment and complex signaling networks to combat fatigue and promote recovery at the cellular level. Acupuncture can regulate the inflammatory and repair microenvironment of skeletal muscles. Exercise, especially eccentric exercise, triggers a local and moderate inflammatory response in skeletal muscles to initiate repair, but excessive or imbalanced inflammation leads to damage and delayed-onset muscle soreness (DOMS). Acupuncture can precisely regulate this process. Studies have shown that acupuncture can inhibit the excessive activation of classic pro-inflammatory signaling pathways such as NF-κB in skeletal muscle tissue, down-regulate the expression of local pro-inflammatory factors (such as TNF-α, IL-1β), and simultaneously up-regulate the expression of anti-inflammatory factors (such as IL-10) and heat shock proteins [34]. This regulatory effect helps to keep the inflammatory response within a range that promotes repair rather than causing damage, accelerating the structural and functional reconstruction of muscle fibers [35]. The restorative and anti-fatigue effects of acupuncture on skeletal muscles are not solely dependent on inflammation regulation; it can also directly affect the structural integrity and functional stability of muscles by regulating protein metabolic balance. Fatigue and overtraining often lead to enhanced catabolism of skeletal muscle proteins, and acupuncture can promote protein synthesis while inhibiting excessive catabolism by regulating key signaling pathways such as Akt/mTOR and FoxO/Atrogin-1. For instance, electroacupuncture intervention has been proven to activate the Akt/mTOR signaling in the skeletal muscles of exhausted rats, up-regulate protein synthesis-related indicators, and inhibit the activity of the ubiquitin-proteasome system, thereby reducing protein degradation in exercise-induced skeletal muscles [36]. This is of great significance for maintaining muscle mass and strength and preventing overtraining syndrome.

## 5. Analysis of the Therapeutic Effects of Acupuncture Therapy on Pre- and Post-Exercise Interventions

The above findings have fully clarified the multi-target and multi-pathway regulatory mechanisms of acupuncture against exercise-induced fatigue, which lays a solid theoretical foundation for its clinical application. Acupuncture’s anti-fatigue efficacy is closely associated with the intervention timing, and its preventive and therapeutic effects are mainly reflected in two clinical intervention modes: pre-acupuncture and post-exercise acupuncture.

### 5.1. Pre-Acupuncture Intervention Before Fatigue

Pre-acupuncture, which involves acupuncture treatment before the occurrence of exercise-induced fatigue, falls within the scope of preventive acupuncture and has the effects of enhancing the body’s immunity, alleviating inflammatory stress responses, and maintaining and repairing mitochondrial energy supply [72]. To evaluate the combined intervention effect based on pre-acupuncture, Miao Jing et al. [44] conducted a randomized controlled study on the combination of acupuncture and massage. The subjects were divided into a control group, a simple massage group, and an acupuncture-massage combination group. By comparing the subjective fatigue scores and serum lactate, lactate dehydrogenase, and creatine kinase levels of the subjects before and after exercise, the results showed that both the acupuncture-massage combination group and the simple massage group could significantly reduce the subjective fatigue scores of the subjects and improve related blood metabolic indicators. Moreover, the combined application of acupuncture and massage was significantly more effective in alleviating exercise-induced fatigue than simple massage treatment. Jiang et al. [45] observed the therapeutic effect of needling Neiguan (PC6) and Zusanli (ST36) on exercise-induced fatigue. Fifty subjects were randomly divided into an observation group and a control group. The observation group received acupuncture at Neiguan and Zusanli before exercise (procedure: insert the needle perpendicularly to a depth of 15–30 mm; upon achieving deqi (Qi sensation), retain the needle for 20 min; after 10 min, apply the ‘neutral tonification and neutral dispersion’ technique once; repeat this once daily for 5 consecutive days), while the control group received no intervention. By comparing the subjective fatigue scores, serum lactate and lactate dehydrogenase levels, heart rate, and blood oxygen saturation of the two groups after exercise, it was found that pre-acupuncture at Neiguan and Zusanli could effectively alleviate exercise-induced fatigue. Hu Weijing et al. [37] used a rat model of exercise to study the effect of different acupoint combinations of pre-acupuncture treatment on the expression of telomerase reverse transcriptase (TERT) in myocardial cells and the release of calcitonin gene-related peptide (CGRP). The acupoints were selected based on the principle of combining the root and the branch, choosing Guanyuan (CV4), which nourishes the innate essence, and ST36, which is the foundation of the acquired essence, along with PC6 to achieve the effect of supporting the body and expelling pathogenic factors. The treatment involved the use of the ‘tonifying and dispersing’ acupuncture technique, with each needle rotated for 1 min and retained for 15 min, over a total treatment period of 6 weeks. The effect was superior to the “health-preserving acupoint” group combining Guanyuan and Zusanli, as well as the blank control group and the exercise group. CGRP is a powerful vasodilator peptide, and its up-regulation helps improve blood oxygen supply to the heart during exercise, which may be one of the important mechanisms by which pre-acupuncture delays overall fatigue. In the study by Shi Junjie et al. [38], transcutaneous electrical acupoint stimulation (TEAS) at the Zusanli acupoint was found to improve cardiac function in a rat model of exercise-induced fatigue. The study randomly divided 21 SD rats into a normal group, a model group, and a TEAS group. After 30 min of TEAS at the Zusanli acupoint (The treatment parameters were as follows: continuous wave, frequency 2 Hz, intensity 5 mA, duration 30 min, until slight tremors were observed in the rat’s limbs), the rats in the TEAS group had a longer time to exhaustion compared to the model group. Additionally, in terms of biochemical indicators, the TEAS group showed an increase in blood UII levels and a decrease in blood BNP levels. This result indicates that TEAS at the Zusanli acupoint can enhance myocardial stress resistance and exert a protective effect on the heart in exercise-induced fatigue.

In summary, the above studies demonstrate that pre-needling treatment before exercise (whether it is simple needling or combined with massage) can enhance the body’s anti-fatigue ability in multiple aspects such as metabolism and cardiac function, highlighting the advantages of the “preventive treatment of disease” concept in traditional Chinese medicine. Pre-acupuncture plays a preventive role by enhancing the body’s anti-fatigue reserve and reducing the degree of exercise-induced fatigue. In contrast, post-exercise acupuncture serves as a remedial intervention, which can effectively alleviate existing fatigue symptoms and accelerate the recovery of physical function in fatigued individuals.

### 5.2. Post-Exercise Acupuncture Treatment

In addition to pre-needling intervention, acupuncture also has a clear improvement and therapeutic effect on already occurred exercise-induced fatigue. A study evaluating the effect of acupuncture on exercise-induced fatigue using transcranial magnetic stimulation randomly divided 20 participants with regular exercise habits into an acupuncture group and a sham acupuncture group. Researchers adopted a two-way repeated measures analysis of variance to compare the effects of acupuncture intervention and time variation on exercise-induced fatigue. The results indicated that needling at ST36 could significantly enhance motor cortex excitability, increase the amplitude of motor evoked potentials (MEP) and shorten MEP latency. It also accelerated the recovery of heart rate to the resting level, which confirms that acupuncture can effectively alleviate exercise-induced fatigue and improve exercise performance [46]. Cardoso et al. [47] conducted a study to evaluate the efficacy of acupuncture on acute muscle soreness (AMS) and delayed onset muscle soreness (DOMS). They randomly divided 45 volunteers into a true acupuncture group, a sham acupuncture group, and a control group. The acupuncture group was treated with needling at the Liangqiu (ST34), ST36, and Taichong (LR3) acupoints. cture group was treated with needling at the Liangqiu, Zusanli, and Taichong acupoints. The muscle soreness and pressure pain threshold at different time points were evaluated in each group. The results showed that the acupuncture group could reduce muscle soreness and improve the pain pressure threshold, indicating that acupuncture therapy has a therapeutic effect on muscle soreness and fatigue caused by exercise. Antonino et al. [48] conducted a three-arm single-blind randomized controlled trial to investigate the effects of traditional Chinese acupuncture and dry needling on peripheral acute fatigue (PAF) of the biceps brachii in untrained healthy volunteers. The study randomly divided 45 volunteers into a traditional Chinese acupuncture group, a dry needling group, and a control group. Muscle fatigue was evaluated through electromyography, VAS, and exercise time. The results showed that the traditional Chinese acupuncture group and the dry needling group had similar temperatures and fatigue sensations, indicating that both therapies have the same effect on body temperature regulation and can improve and restore fatigue.

These findings collectively suggest that post-exercise acupuncture intervention can effectively act on the central (enhancing cortical excitability) and peripheral (relieving muscle soreness) aspects, promoting fatigue elimination and functional recovery of the body.

## 6. Conclusions

In summary, both pre-acupuncture intervention before exercise and post-exercise acupuncture treatment exert significant anti-fatigue effects, and the multi-target regulatory mechanisms of acupuncture against exercise-induced fatigue have been initially clarified, mainly including inhibiting oxidative stress and inflammatory responses, regulating energy metabolism and central nervous system function, improving microcirculation, and modulating skeletal muscle intracellular signaling pathways. Zusanli (ST36) is the core acupoint in most clinical and experimental studies, and different acupoint combinations formulated based on traditional Chinese medicine principles show synergistic anti-fatigue effects, yet a unified and evidence-based standard for acupoint compatibility is still lacking in current research.

Despite the confirmed efficacy and preliminary mechanistic exploration of acupuncture in preventing and treating exercise-induced fatigue, the existing research still has obvious limitations: When classified by study design, current evidence consists of preclinical animal studies and human clinical research. Animal studies dominate the evidence base, whereas clinical evidence is scarce and largely limited to low-quality trials characterized by small sample sizes and single-center designs. Additionally, significant methodological heterogeneity exists across included studies, including inconsistent acupoint selection, variable acupuncture stimulation parameters and non-uniform exercise fatigue modeling protocols in preclinical and clinical research, which impairs the comparability of results. Most mechanistic research remains superficial, and acupuncture operation parameters are not standardized. Therefore, future research should focus on conducting well-designed randomized controlled clinical trials, exploring the optimal acupoint combinations for different populations and exercise types to form standardized protocols, deepening the molecular mechanism research with modern biological technologies, and exploring the synergistic effects of acupuncture combined with other non-pharmaceutical interventions. The cross-integration of multiple disciplines will further promote the standardized clinical application and development of acupuncture in the field of sports medicine.

## Figures and Tables

**Table 1:** **Table of Characteristics of Included Basic Experimental Studies.**

No.	Author, Year	Study Type	Exp. Subject	Intervention	Key Outcome
[14]	Ma H.F. et al., 2015	Animal Exp.	Male SD rats	Acupuncture at SP6+ST36/BL23+ST36/GV14+ST36	SOD ↑; GSH-Px ↑; MDA ↓
[15]	Pejon T.M.M. et al., 2025	Animal Exp.	SD rats	Melatonin (10 mg/kg, ip) 30 min pre-model	Carbonylated protein ↓; lipid peroxidation↓; muscle oxidative damage ↓
[16]	Yang Y.Q. et al., 2024	Animal Exp.	Male SD rats	Acupuncture at ST36+CV12 (10 min/d × 7 d)	SOD ↑; GSH-Px ↑; MDA ↓; TNF-α ↓; IL-1β ↓; IL-6 ↓; Nrf2 ↑
[17]	Liu S. et al., 2021	Animal Exp.	C57BL/6 mice	Low-intensity EA at ST36	Vagal-adrenal axis ↑; systemic inflammation ↓; PROKR2+ neurons mediate effect
[18]	Bai S.C. et al., 2020	Animal Exp.	Male SD rats	Acupuncture intervention	DRP1 ↓; FUNDC1/DRP1 ↓; mitochondrial fragmentation ↓
[19]	Li C.L. et al., 2024	Animal Exp.	SD rats	EA at GV20+GV14 (20 min/d × 7 d)	Drp1 ↓; Fis1 ↓; Mfn1/2 ↑; OPA1 ↑; neurological deficit ↓
[20]	Lou H. et al., 2024	Animal Exp.	SD rats	Acupuncture intervention	PGC-1α ↑; NRF1 ↑; TFAM ↑; ATP ↑; SOD ↑; MDA ↓
[21]	Wu J. et al., 2017	Animal Exp.	SD rats	EA at LI11+ST36 (1/d × 7 d)	AMPKα phosphorylation ↑; cerebral glucose metabolism ↑; infarct volume ↓
[22]	Cai J.M. et al., 2024	Molecular Exp.	Mice	XHY69AP (100/200/400 mg/kg/d, ig × 4 w)	Exhaustion time ↑; glycogen/ATP ↑; lactic acid/BUN/CK/LDH ↓; SOD/GSH-Px ↑; AMPK/PGC-1α ↑
[23]	Hadzimustafic N. et al., 2020	Cell Exp.	Human primary myotubes	AMPK inhibitor; glycogen phosphorylase inhibitor	OCR ↑; ECAR ↓; FAO ↑; mitochondrial fusion ↑; mTOR ↓; autophagy/mitophagy ↑
[24]	Bardgett M.E. et al., 2009	Animal Exp.	SD rats	Dopamine receptor agonist/antagonist	D1/D2 mediate effort-based decision-making
[25]	Li H.L. et al., 2015	Animal Exp.	SD rats	Manual/EA/warm acupuncture at ST36	Exhaustion time ↑; hypothalamic 5-HT ↓; 5-HIAA/5-HT ↑
[26]	Fang J.Q. et al., 2012	Animal Exp.	SD rats	TEAS	Hippocampal/hypothalamic 5-HT ↓; neuronal damage ↓; fatigue recovery ↑
[27]	Yang Y. et al., 2016	Animal Exp.	SD rats	EA at ST36+LR3 (2 Hz/100 Hz, 2 mA, 30 min/d × 14 d)	Gastric emptying ↑; intestinal propulsion ↑; NT ↓
[28]	Yang Y. et al., 2018	Animal Exp.	SD rats	EA at GV20+Ningshen+bilateral sensory areas	Open-field activity ↑; hippocampal/hypothalamic neuronal damage ↓
[29]	Su Y. et al., 2022	Animal Exp.	SD rats	EA at PC6	Sympathetic activity ↓; vagal activity ↑; autonomic balance restored; HR/BP regulated
[30]	Ji G.C. et al., 1996	Animal Exp.	SD rats	EA at LI11/ST36/GV26+PC6	Cerebral ischemic microcirculation blood flow ↑
[31]	Guo J. et al., 2025	Animal Exp.	Male SD rats	Acupuncture at GV26+GV20+GB20+ST36 (1/d × 14 d)	Cognitive function ↑; CKLF1/CCR5 ↓; HIF-1α ↓; cerebrovascular damage ↓
[32]	Ma Y.N. et al., 2007	Animal Exp.	SD rats	Medicine-separated moxibustion	Blood viscosity ↓; erythrocyte rheology improved; exercise capacity ↑
[33]	Langevin H.M. et al., 2006	Animal Exp.	Mice	Acupuncture needle rotation	Fibroblast cytoskeletal remodeling ↑; myofascial tension ↓; microcirculation ↑
[34]	Li H. et al., 2024	Animal Exp.	Male SD rats	Acupuncture pretreatment	cGAS-STING-NF-κB ↓; IL-8 ↓; IFN-β ↓; skeletal muscle mitochondrial damage ↓
[35]	Huang Y.T. et al., 2025	Animal Exp.	SD rats	EA at BL57+GB34 (2 Hz/100 Hz, 2 mA, 30 min/d)	Macrophage M2 polarization ↑; satellite cell proliferation ↑; TNF-α/IL-1β ↓
[36]	Huang Y.T. et al., 2024	Animal Exp.	SD rats	EA at BL57+GB34 (+sodium cromoglycate)	Mast cell degranulation ↑; 5-HT ↑; Pax7/MyoD ↑; CD206 ↑; CD68 ↓
[37]	Hu W.J. et al., 2018	Animal Exp.	SD rats	Acupuncture pretreatment	Myocardial mitochondrial ND1 ↓; TERT ↓; CGRP ↑; myocardial oxidative damage ↓
[38]	Shi J.J. et al., 2020	Animal Exp.	SD rats	TEAS at ST36 (2 Hz, 5 mA, 30 min pre)	Exhaustion time ↑; serum BNP ↓; serum UII ↑

Note: ↑ indicates upregulation/increase; ↓ indicates downregulation/decrease.

**Table 2:** **Table of Characteristics of Included Clinical Experimental Studies.**

No.	Author, Year	Study Type	Sample Size (*n*)		Intervention Methods		Acupoint
			Experimental Group	Control Group	Experimental Group	Control Group	
[39]	Komine S et al., 2025	RCT	9	10	Ipsilateral electroacupuncture	Contralateral electroacupuncture	/
[40]	Li Z. X. et al., 2022	RCT	36	36	Electroacupuncture on the back	Fake electroacupuncture on the back	BL18, BL15, BL20, BL13, BL23
[41]	Shu Q et al., 2016	RCT	30	15	Manipulation acupuncture (15)/indirect moxibustion (15)	Rest	ST36, CV4
[42]	Djaali W et al., 2023	RCT	12	12	Acupuncture before high-intensity exercise	High-intensity exercise	ST36, PC6
[43]	Xiong J et al., 2024	Systematic Review	427	425	Direct acupuncture (291)/acupuncture and massage (136)	medication for oral treatment (227)/rehabilitation (30)/lidocaine injections (48)/McKenzie therapy (30)/herbal medicine (90)	ST36
[44]	Miao J. et al., 2020	RCT	80	40	Acupuncture and Tuina Combination Group (40)/Tuina Group (40)	Rest	BL23, BL57
[45]	JIANG T et al., 2019	RCT	25	25	Acupuncture	Rest	ST36, PC6
[46]	HU L et al., 2024	RCT	10	10	Acupuncture	Sham acupuncture	ST36
[47]	CARDOSO R et al., 2020	RCT	15	15	Verum acupuncture	Rest	ST36, ST34, LR3
[48]	ANTONINO G et al., 2023	RCT	15	15	Dry needling	Rest	LI1, TH5, LI10, LI11, LI14, GB21

## Data Availability

No new data were created or analyzed in this study. Data sharing is not applicable to this article.

## Abbreviations

The following abbreviations are used in this manuscript:

Akt/mTOR	Protein kinase B/mammalian target of rapamycin
AMPK	Adenosine 5′-monophosphate-activated protein kinase
CGRP	Calcitonin gene-related peptide
cGAS-STING	Cyclic GMP-AMP synthase-stimulator of interferon genes
deqi	Qi sensation
DOMS	Delayed-onset muscle soreness
DRP1	Dynamin-related protein 1
EIF	Exercise-induced fatigue
FoxO/Atrogin-1	Forkhead box O/Atrogin-1
FUNDC1	FUN14 domain-containing protein 1
GSH-Px	Glutathione peroxidase
HSP	Heat shock protein
HIF-1α	Hypoxia-inducible factor 1α
HRV	Heart Rate Variability
IL	Interleukin
LDH	Lactate dehydrogenase
MEP	Motor evoked potential
MDA	Malondialdehyde
MFN1	Mitofusin 1
NF-κB	Nuclear factor-kappa B
OPA1	Optic atrophy 1
PGC-1α	Peroxisome proliferator-activated receptor γ coactivator 1α
RCT	Randomized controlled trial
RMT	Resting motor threshold
RNS	Reactive nitrogen species
ROS	Reactive oxygen species
RPE	Perceived exertion
SF-36	36-Item Short Form Health Survey
FS-14	Fatigue Scale-14
SOD	Superoxide dismutase
TEAS	Transcutaneous electrical acupoint stimulation
TNF-α	Tumor Necrosis Factor-α
TOMM20	Translocase of outer mitochondrial membrane 20
VEGF	Vascular Endothelial Growth Factor
